
JOURNAL INFORMATION
==============================
NLM Title Abbreviation: Med Chir Rev
NLM Journal ID: 0034617

The Medico-Chirurgical Review

Publisher: Biomedical Journal Digitization Project

ARTICLE INFORMATION
==============================
PMCID: PMC4966266
PMID: 29917636
Article version: 1
Subjects: Articles

Hospital Practice

Print publication date: 1828 Oct 1
Volume: 9
Issue: 18
First page: 525
Last page: 528
1828 Aug
License: This work is free of known copyright restrictions. For more information, please see PMC Back Issue Digitization.
License URL: https://creativecommons.org/publicdomain/mark/1.0/

==============================

gradation which, as a licentiate, he must undergo, if he settles in the metropolis, or within seven miles thereof. This information should be pondered on by parents!

### Smoking Tobacco in Hernia.

In a recent journal of this metropolis, there is a serio-ludicrous proposal from Mr. Wallace, of the Royal Navy, respecting the superiority of smoking tobacco over the same drug used as an enema. It is probable that Mr. Wallace has not accurately observed the effects of tobacco given per anum. The death-like coldness and pallor, the feeble pulse, the relaxation of the muscles, and the tendency to syncope, are independent of sickness or vomiting, and precede that operation. These phenomena are totally different from that nausea which is produced by smoking tobacco for the first time. We can assure Mr. Wallace that his plan would be entirely fruitless, and would, in no efficient degree, contribute to the reduction of a hernial protrusion.

### HOSPITAL PRACTICE.

### 61. Royal Infirmary of Glasgow.

A quarterly report of the cases in the surgical wards of the above institution, is published by Dr. Anderson, in the third Number of the Glasgow Journal. Before he proceeds to particular cases, the Doctor has given a table of maladies admitted, and operations performed, shewing, in the gross, the surgical practice of the hospital.

### I. Artificial Anus treated by compression and the Cautery.

Baron Dupuytren's memoir, of which we have given so ample an account, will make, if we mistake not, an important improvement in the cases of artificial anus occurring in consequence of hernia or wounds. The subject of the present case was a Mary Stewart, admitted on the 10th November, with a very large sore in the left groin, extending from the spine of the ilium to the symphysis pubis. It was three inches broad, passed above Poupart's ligament, was ragged, irregular and sloughy, and a sinus extended from it down by the side of the labium, from which could be pressed a good deal of purulent matter. The gut protruded as a round firm substance in the centre of the sore, and immediately above the protrusion was seated the opening, through which all the fæces were evacuated. The poor girl was hectic and greatly emaciated, and the pulse was so frequent and feeble as not to admit of being counted. For twelve months she had been subject to femoral hernia, which, a month before admission, had apparently got strangulated, and sloughed in eight days, from which time the fæces had passed by the wound.

"This case was very unpromising, and from the excessive debility, it seemed almost hopeless. A nourishing diet, with a liberal allowance of wine and brandy, produced little improvement. The appearance of the discharges soon showed that her debility chiefly arose from inanition, owing to the artificial opening having taken place in a part of the gut where the process of assimilation was still incomplete. With the double purpose, therefore, of affording nourishment, and dilating the lower intestines, I directed the use of large beef-tea enemata three times a-day.

"The next object was to prevent the escape of the contents of the gut, which not only kept up the debility, but produced sloughing of the sore, and hindered it from healing. The aperture admitted the finger, by which it was ascertained that only one side of the gut had been destroyed, and that the other side formed a continuous surface towards the abdomen. This case, therefore, did not require the application of the ingenious instrument of Baron Dupuytren. Plugging the opening with various conical shaped sponges was first tried; but she could not bear the degree of pressure necessary to retain them *in situ*. Recourse was therefore had to a long cylindrical tent of lint firmly rolled up.

This was pushed deeply into the gut, so as completely to fill the external aperture, and in this way nearly to maintain itself in position. Over this graduated compresses were placed, and the whole supported by a light truss. She could not wear any kind of truss, however, more than a few hours at a time, and firm bandaging was substituted for it." 314.

The next day flatus was discharged from the rectum, and natural evacuations succeeded, and continued throughout the remainder of the treatment. Chloride of lime and lunar caustic were applied, and the sore, at the end of December, had contracted to the size of a shilling. The objection to the tent was its resting in the gut, obstructing the flow of the fæces in the tube, and hindering the cicatrization of the sore by forcing a portion of the contents of the intestine outward by the wound. An instrument was accordingly invented, that should not be attended by the same inconvenience, but it failed and was finally abandoned.

On the 16th of January, the opening was touched with the actual cautery, which checked all discharge for several days, and produced a decided contraction, after the separation of the slough. The cautery was frequently repeated, and under its employment the fistulous opening diminished to a very small point: the discharge was but trifling and occasional only: the health was revived; and, expressing a wish to go home, she left the infirmary at the latter end of April. The fistula was trifling, but the smaller it is the greater in general the difficulty of closing it completely, though productive of little inconvenience. With respect to the cautery, Dr. Anderson remarks, that he has found it very serviceable in many cases.

"I have cured several fistulæ of the urethra in this way, which had resisted every other means; and I lately saw a patient, from whom I removed a very large fungus of the antrum five years ago. Her disease has not returned, solely, I am convinced, owing to the free application of the hot iron. In hæmorrhagic oozings from sores or wounds, by which debilitated patients are frequently cut off, the eschar formed in this way is very effectual; and in obstinate bleeding from leech-bites, especially in restless children, where pressure is difficult, the point of a red hot wire will at once check the discharge." 315.

That the pain and inconvenience of the actual cautery have been greatly exaggerated, we have very little doubt, but the prejudices against it even on the part of the patient, are often insuperable, generally strong. In Dupuytren's cases, that celebrated surgeon pared the callous edges of the wound, and brought the raw surfaces together by means of compression and straps. We wonder the above was not adopted in the present case, as Dr. A. would appear to have been aware of the practice of the Frenchman.

### II. Dislocations of the Clavicle.

Every body knows the difficulty frequently experienced in managing dislocations and fractures of the clavicle. Sir Astley Cooper observes, that "it very rarely happens that these accidents to the clavicle are perfectly recovered from, some degree of deformity usually remaining. Of this the patient should always be informed at the commencement of the treatment, as otherwise he may attribute it to the negligence or ignorance of the surgeon."

Dr. Anderson conceives that the tendency to displacement after reduction, which is infinitely greater in some cases than in others, depends on the greater or less degree of injury done to the ligaments, particularly those between the coracoid process of the scapula and clavicle. The bandage of Brasdor, represented in the Mémoires de l'Académie de Chirurgie, and similar in principle to that of Sir A. Cooper, is probably the best for the purpose. Dr. Anderson, however, has found that confinement on the back for a fortnight, with a pad in the axilla, and the elbow advanced and bound to the thorax is preferable to any kind of bandage. "A narrow firm pillow placed between the scapulæ, will likewise conduce to throw back the shoulders, without exciting any opposing action in the anterior muscles." The difficulty is in so trifling an accident to prevail on a patient to submit to the confinement to bed.

One of the cases of luxation of the humerus into the axilla, was accompanied with a curious fracture of the bone. The dislocation was reduced, but the man died next day, from the effects of other injuries. On examining the joint, it was discovered that the portion of the greater tubercle of the humerus, to which the tendon of the supra-spinatus is attached, was fractured and detached. Part of it being within, and part on the outside of the capsular ligament, it would have been a matter of interest, of course, to have seen whether union would have taken place. Dr. Anderson believes that it would, and we think it exceedingly probable.

### III. Fatal Case of Suppuration beneath the Fascia of the Fore-arm.

Mary Drysdale, æt. 35, was lifting a heavy weight when she sprained her left wrist; and on the 1st of March, eight days after the occurrence of the accident, she was admitted into the Royal Infirmary. The two lower thirds of the arm, especially in front, were much swollen; the hand also swollen, and the back of it œdematous; the skin of a bright red colour, glossy, and tense; the pain so severe as to hinder her getting any sleep; the pulse 110; bowels costive; tongue furred, with head-ache and anxiety of countenance.

A purgative was given, and an incision immediately made on the front of the fore-arm. Another incision was made in the direction of the ulna on the 2d, and, on the 3d, the arm was easier, and the tension diminished. She had not, however, obtained much relief on the whole, and there being much heat and pain about the elbow, 12 leeches were applied, and a saturnine lotion. On the 4th, the whole arm was less inflamed, but the tension and pain had increased in the back of the hand. Suppuration took place in the palm, and incisions were made on the 6th, about the thumb and little finger, and a bandage was afterwards applied. The relief was so trifling, that Dr. Anderson was led to suspect the existence of matter beneath the aponeurosis of the deep flexor muscles. On examination, obscure fluctuation was detected, and an incision carried deeply midway between the elbow and wrist, which gave issue to a mixture of purulent matter and sloughs. In this incision, an artery of magnitude was divided, and bled profusely beneath the fascia, which was instantly distended. The vascular condition of the parts, the depth of the vessel, and debilitated state of the patient, deterred Dr. A. from extending the incision, and securing the vessel at the wound. Accordingly, the humeral artery was tied above the elbow, whilst the wound in the fore-arm was afterwards enlarged for the free discharge of the coagula and pus.

The suppuration now became profuse; the ring and little finger, having sphacelated, were obliged to be removed; the thumb became livid, but gradually recovered, and the patient expired on the night of the 14th of March.

The ligature had separated from the humeral artery, which was closed, and the wound nearly healed. The vessel which was wounded could not be discovered, but Dr. Anderson thinks it was probably the interosseous. The muscles and soft parts had put on a healthy appearance, and the patient seemed to sink from the exhaustion produced by the discharge.

This case, when conjoined with the one that occurred to Mr. Lawrence, should teach a little caution in the practice of incisions. We should try to evacuate matter without evacuating at the same time a pint or two of blood.

---

### 62. Penetrating Wounds of the Abdomen, with Issue of the Viscera.

[Hôpital de la Garde Royale.]

*Case* 1. A young soldier received, in a duel, a thrust from a sabre on the anterior part of the abdomen, a little above and to the right of the umbilicus. The abdominal parietes were divided, and a considerable mass of omentum protruded from the belly. The patient was removed to the hospital, and attempts having been made, without success, to reduce the protruding omentum, some linen, spread with simple dressing, was applied upon it, and every precaution put in force to prevent the occurrence of peritoneal inflammation. Bleedings from the arm—applications of leeches to the epigastrium—cupping and fomentations to the abdomen, and a rigorous diet, were the principal means, and effectually checked the inflammatory symptoms.

Eight days after the infliction of the injury, the omentum was cut off. The surface, which was cut, furnished healthy granulations, which were touched now and then with the actual cautery—no pain nor sensation of dragging was experienced—the functions of digestion were as regular as ever, and at the end of five weeks the patient was very nearly well.

*Case* 2. A horse-soldier, whilst engaged in a duel, received the point of his adversary's weapon in the right groin, a little to the inside of the anterior superior spinous process of the ilium. The Fallopian ligament was cut across, and a portion of the small intestine issued from the wound, which the surgeon of the corps contrived to reduce, by enlarging the opening through which it had passed. Some hæmorrhage occurred, but none of any consequence, and the patient was bled from the arm, leeched, cupped in the loins and hypochondrium, kept upon a very rigid diet, and every measure had recourse to, which was likely to ward off peritoneal inflammation.

This latter did not take place, but the edges of the wound became swollen and inflamed, and considerable suppuration was the consequence. A counter-opening was required along the margin of the rectus muscle, and a second on the 30th of April, rather higher than the former. The patient was doing well, and was expected to be speedily cured.—*Clinique.*

It has always been a matter of surprise among surgeons, that the abdominal viscera should escape as they do. In the cases recorded above, the wounds were of a nature very likely to injure the intestines, and yet they got off in both instances scot-free. A very interesting case is related in his lectures by Sir Astley Cooper, where, in spite of the infliction of several penetrating wounds of the abdomen, not a single bad symptom supervened. Sir Astley likewise mentions a case, that is strikingly illustrative of the manner in which the intestines glide away from the edge of the instrument.

*Case* 3. Sir Astley was called to a house in the Borough, and shewn into a room, where he found a female in her shift, lying on the floor, weltering in her blood. Having raised her with difficulty, and placed her on the bed she had just quitted, four wounds were discovered in her throat, one of which was deep and extensive.

"These I closed by sutures; after which she was able to speak, and I then asked her what had induced her to commit the act? She made an incoherent reply, but repeated the word stomach two or three times, which induced me to raise her linen, when I was surprized to find her bowels exposed by a wound reaching nearly from the pubes to the ensiform cartilage of the sternum; for, after cutting her throat with a razor, she had ripped up her belly with it, and let out her bowels, but the intestines were still distended with air, and I had a difficulty in returning them into the abdomen. They had not received the smallest wound: Dr. Key now came into the room, and I proceeded to sew up this extensive opening, but she died in nine hours."

The mode of treatment which Sir Astley recommends is as follows:—

"In the treatment of these wounds it is best to make interrupted sutures; the needle should penetrate the skin and the muscles, but not the peritoneum. If the muscle be not included in the ligature, a hernia is sure afterwards to form; and, if the thread is introduced through the peritoneum, it adds much to the danger of peritoneal inflammation.

"Between the sutures, strips of plaister, or of lint dipped in blood, should be applied, and the patient should be freely bled from the arm. If the local inflammation be great, leeches should be employed; purgatives must be avoided, and food must not be given for several days."

We think Sir Astley might have dwelt with greater force upon local depletion. The abdomen should be covered with leeches, and we are sure that in cases we have witnessed, particularly of peritoneal inflammation after hernia, this measure has been greatly neglected, though a powerful adjuvant to general bleeding.

If the stomach or intestines are wounded, the danger, of course, is incalculably greater. Even, however, in cases like these, the recoveries are often astonishing, and calculated to shew the restorative powers which Nature can exert at a push. The case of Mr. Scott, where the patient survived a severe wound of the stomach, as well as the one in Baron Dupuytren's Memoir, where a maniac cut away half a foot of small intestine, are very remarkable instances of this kind.
